# Lab-on-a-Chip for Cardiovascular Physiology and Pathology

**DOI:** 10.3390/mi11100898

**Published:** 2020-09-28

**Authors:** Sean Beverung, Jingwen Wu, Robert Steward

**Affiliations:** Department of Mechanical and Aerospace Engineering, Burnett School of Biomedical Sciences, University of Central Florida, Orlando, FL 32816, USA; K214beverungs@knights.ucf.edu (S.B.); Jingwu@Knights.ucf.edu (J.W.)

**Keywords:** lab-on-a-chip, cardiovascular disease, microfluidics, cell culture

## Abstract

Lab-on-a-chip technologies have allowed researchers to acquire a flexible, yet relatively inexpensive testbed to study one of the leading causes of death worldwide, cardiovascular disease. Cardiovascular diseases, such as peripheral artery disease, arteriosclerosis, and aortic stenosis, for example, have all been studied by lab-on-a-chip technologies. These technologies allow for the integration of mammalian cells into functional structures that mimic vital organs with geometries comparable to those found in vivo. For this review, we focus on microdevices that have been developed to study cardiovascular physiology and pathology. With these technologies, researchers can better understand the electrical–biomechanical properties unique to cardiomyocytes and better stimulate and understand the influence of blood flow on the human vasculature. Such studies have helped increase our understanding of many cardiovascular diseases in general; as such, we present here a review of the current state of the field and potential for the future.

## 1. Introduction

Lab-on-a-chip is a small flexible platform that allows for the design and execution of specialized experiments in a countless number of fields. The platform has been adopted for use in the biomedical community as a tool that allows studies to use a human model when combined with tissue culture techniques [1,2,3,4,5,6]. These specialized platforms have been made to simulate different aspects of the cardiovascular system. Two examples of this are heart-on-a-chip and vascular microchannel chips [1,4,7,8]. With these specialized chips, disease conditions can be simulated and used to gain a better understanding of the cardiovascular system and its diseases and test the effectiveness of medications [1,2,4,7,8,9,10,11]. Several microfabrication techniques are commonly employed to create chips that support cell culture and simulate a disease condition of interest. In this review, we will look at the cardiovascular diseases of atherosclerosis, cardiac fibrosis, and thrombosis. After this, we will discuss common fabrication techniques to produce lab-on-a-chip platforms that simulate or are used to gather data on these diseases. Lastly, we will look at how lab-on-a-chip is used to collect data on the effects of medications, the electromechanics of the heart, and the impacts of altered fluid flow due to stenosis and thrombosis on the cardiovascular system.

## 2. Cardiovascular Disease

**Figure 1:**
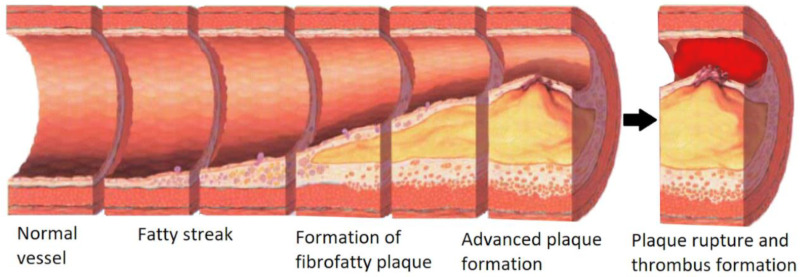
From left to right, Normal healthy vessel, Fatty streak formation, the formation of fibrofatty plaque into an advanced unstable plaque narrowing the lumen of the blood vessel. Then unstable plaque rupture and thrombosis. Adapted from Nicholas Patchett under CC BY-SA 4.0.

Cardiovascular disease is among the leading causes of death in the United States and worldwide. One type of cardiovascular disease, coronary artery disease, is caused by a restriction of blood flow through the coronary artery [12,13,14,15]. The most common way for this blockage to occur is from the build-up of cholesterol-rich fibrofatty plaque on the inside of blood vessels through a process called atherosclerosis [16,17,18]. Atherosclerosis can cause stenosis, a narrowing of the blood vessels. In this way, atherosclerosis can reduce or in severe cases, completely block blood flow to the heart leading to inadequate oxygen supply for the heart′s metabolic function (ischemia) [19,20]. This lack of oxygen can cause an irregular and potentially deadly contractile response of the heart′s muscle tissue or heart arrhythmia. Furthermore, a more substantial reduction in oxygen can lead to tissue death, resulting in a myocardial infarction or a heart [20,21,22]. In addition, atherosclerosis can influence the blood flow in the affected region, increasing hemodynamic forces on the vessel walls and further affecting vascular health in a deleterious manner. The increase in hemodynamic forces can also increase the likelihood of the formation of a blood clot, also known as a thrombus. This thrombus can break free from the vessel wall and block blood flow within a narrower region of the vasculature further downstream, potentially leading to a stroke or myocardial infarction, depending on where the thrombus ends up. Hemodynamic forces also influence endothelial cell gene expression [17,23,24]. Under certain conditions, plaque build-up within the interior of the blood vessel can rupture due to mechanical weakening of the endothelium, increasing the chance of blood clot formation [17,25,26] (Figure 1 [27]). Another common cardiovascular disease is cardiac fibrosis. Cardiac fibrosis is a condition that describes an increase in the proliferation of cardiac fibroblasts and the increased deposition of extracellular matrix by these fibroblasts [19]. This excess extracellular matrix leads to increased stiffening of the heart tissue and can lead to dysfunction of the heart tissue in a multitude of ways. For example, fibrosis can impede the electrical signals in the heart, slowing them down and causing irregular patterns of conduction [28]. Fibrosis can also cause stiffening of the heart valves, which can lead to an inability for the valves to open and close properly. The valve′s failure to fully function can cause regurgitation of blood into the heart chamber, reducing the heart′s blood output [29,30].

To better study the diseases mentioned above, both in vivo and in vitro experiments were utilized. Although in vivo experimental setups can generally be performed utilizing living organisms or ex vivo using whole tissue models, in vitro experiment designs typically consist of monocellular or multicellular cultures [31,32,33,34]. For cardiovascular disease-on-a-chip applications, such a setup may include cardiomyocytes, cardiac fibroblasts, and endothelial cells, for example. While both in vivo and in vitro studies each have their unique pros and cons. In vitro technologies such as lab-on-a-chip have a unique advantage in the fact that multiple experimental testing devices can be mass fabricated in a relatively short amount of time at a lower cost when compared to in vivo [35,36,37,38]. Pathology-on-a-chip technologies can also be used to determine the individual contribution of specialized cells to cardiovascular physiology and pathology as well.

Cardiomyocytes, for example, are used in studies directly relating to heart muscle tissue as they are the cell responsible for the heart′s ability to contract and pump blood to compose the primary cell mass of the heart [39,40,41]. The pulsatile nature and electrical sensitivity that the cardiomyocytes exhibit can be monitored through lab-on-a-chip studies. Cardiac fibroblasts also play an important part in the function of the heart as they are the most common cell type in the heart but occupy much less volume than cardiomyocytes. Cardiac fibroblasts create the ECM that supports the heart tissue [41]. Cardiomyocytes can be co-cultured with cardiac fibroblasts for studies that require the simulation of cardiac fibrosis [31]. Another type of cardiovascular cell type that is used and highly popular in the field are endothelial cells. Endothelial cells line the inside of all blood and lymphatic vessels within the body and play an essential role in the regulation of material in and out of the blood vessel. Utilizing the cells mentioned above, one can develop a customized cardiovascular disease-on-a-chip device to simulate the conditions of specific regions of the cardiovascular system and specific cardiovascular diseases. Cardiovascular pathology-on-a-chip platforms can be fabricated in several ways, each with its advantages and disadvantages. These technologies offer more of a controlled and customized testing platform that just would not be available otherwise. Below, we present cutting edge microfabrication technologies, the construction materials commonly used with the fabrication technology, and examples of cardiovascular disease-on-a-chip devices that were fabricated with these techniques and their application in studying various heart conditions.

## 3. Lab-on-a-Chip Microfabrication Methods

### 3.1. Photolithography

**Figure 2:**
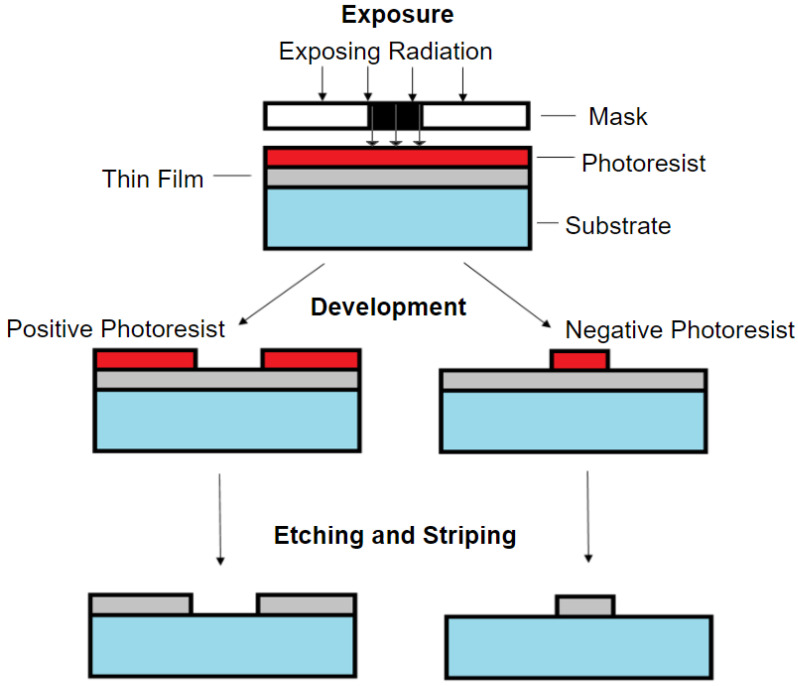
Process of photolithography. Initially, the film of photoresist is coated on the substrate, and photoradiation is used to cure a region coated by the photoresist (the curved region is removed with a positive photoresist remover and the uncured region is removed with a negative photoresist remover).

One of the most common microfabrication methods used for lab-on-a-chip is photolithography. Explaining the process involved with photolithography, as shown in Figure 2: photolithography uses UV light to transfer the predesigned pattern from a photomask to a substrate or thin film [42]. First, the substrate will need to be cleaned and subsequently coated with a photosensitive chemical called photoresist. Next, a mask with a predesigned pattern is suspended over the photoresist, and UV light is used to cause a chemical reaction in the photoresist. Depending on the photoresist, a positive or negative pattern is formed when the photoresist is developed. This is because there are two types of photoresists: positive and negative, which react differently when exposed to the UV light and whose use will depend on the intended structure that is to be fabricated [42,43]. Positive photoresist becomes more soluble and is removed after exposure to UV light, while negative photoresist polymerizes, hardens, and remains after exposure to UV light. Regardless of the photoresist used, the UV light will transfer a near-identical copy of the pattern from the predesigned photomask on the photoresist-coated substrate. However, in general, positive photoresists have higher resolution capability, which has better stability to maintain the pattern size [44]. In addition, positive photoresist requires higher resolution, the photo coating speed will be lower, and the operation cost will be higher compared to the negative photoresist used [44]. The hardened photoresist can be used as a template for soft lithography. Additionally, etching or stripping can be used to leave behind a thin film of material that can be used to make electrodes. The low-cost and relatively fast ramp-up time for photolithography has provided an advantage to fabricate lab-on-a-chip devices and displays why this method has been and will continue to be a popular method in the field. Photolithography allows for the construction of microchannels and other microfluidics that provide the ability to replicate structures found in vivo such as blood vessels. With the capability of analyzing the influences of the different blood flows/shear rates could be involved in the progression for cardiovascular diseases developments [45,46].

### 3.2. Soft Lithography

**Figure 3:**
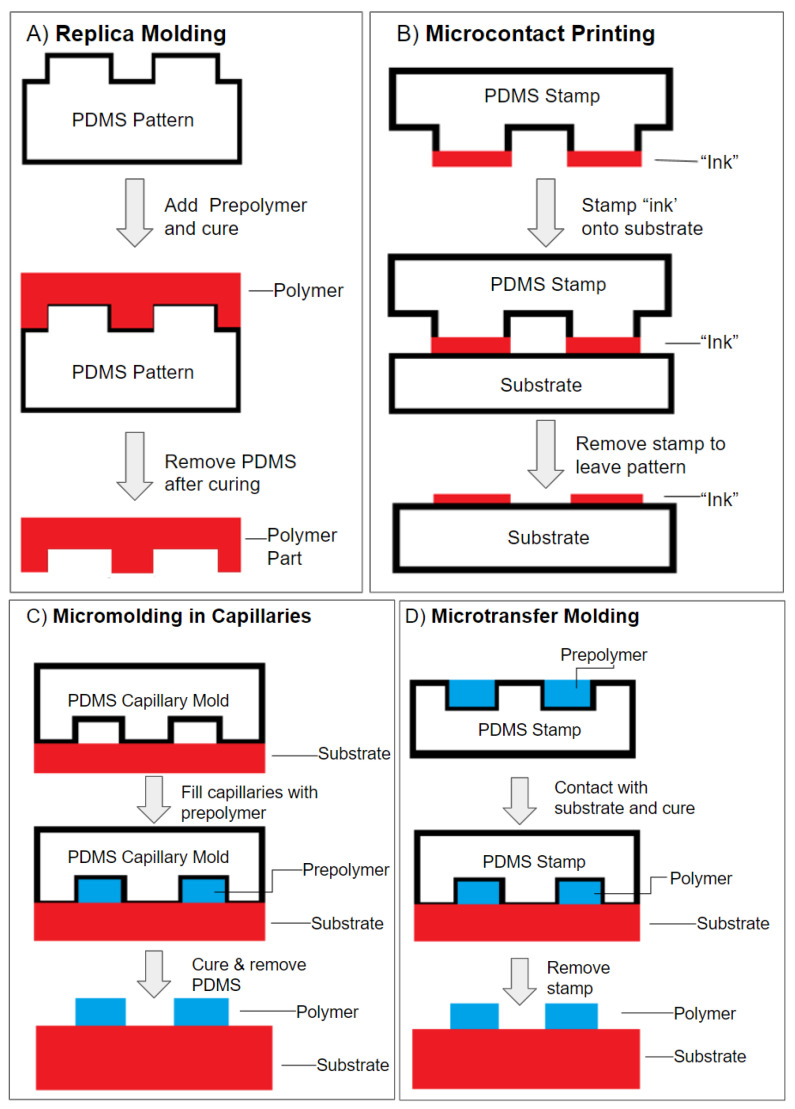
The four core techniques of soft lithography. (**A**) Replica molding. (**B**) Microcontact printing. (**C**) Micromolding in capillaries. (**D**) Microtransfer molding.

Soft lithography is another popular fabrication method used in lab-on-a-chip. This technology allows the fabrication of predesigned microstructures using various designs of molds, photomasks, and stamps [47]. Soft lithography also can be viewed as a complementary technique for photolithography as it can be used as a template to fabricate geometrically complex structures with biocompatible materials, allowing the creation of biological systems for cell/tissue growth [48]. The common material used in soft lithography is polydimethylsiloxane (PDMS), which can be classified into four core techniques: (1) Replica molding: simply duplicated the required structures by just pouring the PDMS on the predesigned pattern and cured (Figure 3A) [6,36,37,49,50,51,52]. (2) Microcontact printing: used the PDMS pattern as a stamp to transfer the molecular “ink” to the surface of the substrate, allowing the creation of patterns on porous surfaces (Figure 3B) [6,36,53,54]. (3) Capillary molding: replicated the structure by drawing the liquid polymer through capillary action to the boned PDMS pattern and substrate (Figure 3C) [6,55]. (4) Microtransfer molding: used the PDMS pattern as mold and filled with other liquid polymers. This method provides a reusable template with the advantage of being able to produce structures with small dimensions (Figure 3D) [6,56]. In general, the use of soft lithography techniques has provided the ability to replicate the predesigned microstructures using four core techniques, and biopolymers used have provided a biocompatible environment for cell culture. These biopolymers aid in the simulation of in vivo structures and study associated pathologies.

### 3.3. 3D Printing

3D printing has become a popular method recently used to fabricate the cardiovascular lab-on-a-chip devices using in vivo geometries in a convenient way by (1) fabricating a complete lab-on-a-chip device in a single step from a computer model within the required device specifications and (2) allowing researchers to closely mimic the complex organ and tissue geometries found within the human body gained from various imaging modalities. Another benefit of 3D printing is the reduced fabrication time and errors when compared to photolithography and soft lithography [35]. There are two types of 3D printing methods: traditional and bioprinting. Traditional 3d printing uses conventional materials such as plastics to construct a device.

**Figure 4:**
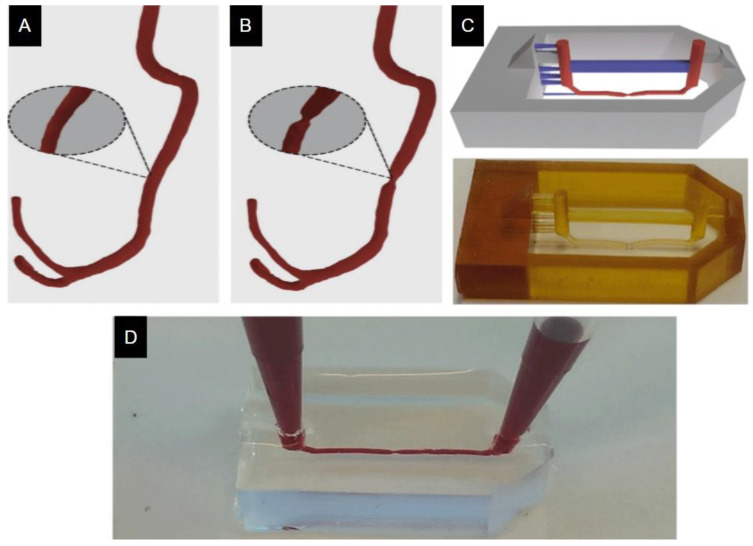
Three-dimensional models for (**A**) healthy and (**B**) stenosis vessel. (**C**) Top: Image of the 3D model; in grey are the outer walls of the mold, in blue are the printed support structures, and in red are the microfluidic channels. Bottom: Image of the photography of the printed model. (**D**) the complete polydimethylsiloxane (PDMS)-casted microfluidic chip after mold removal. Adapted from Costa et al. [21].

An example of a device created with traditional 3D printing is one created by Costa et al. using stereolithography (SLA) 3D printing. This method was used to construct microdevices containing miniaturized vascular structures that mimicked healthy and stenotic blood vessels [21]. The design of stenosis condition was printed as templates on stiff substrates and used as molds for PDMS-based soft lithography to replicate the in vivo vessel geometries observed during stenosis and thrombosis conditions (Figure 4). This new approach has helped advance the field as it allows for recapitulation of the architecture found in both physiological and pathological conditions with high precision by using the data obtained from Computed Tomography Angiography (CTA) [57,58]. This technology can be used for further study of cardiovascular drug screening.

In addition to the traditional 3D printing method mentioned above, 3D bioprinting has become another popular technology due to the excellent biocompatibility compared to the other fabrication methods described above. Three-dimensional bioprinting can produce biomimetic structures by depositing living cells layer-by-layer to generate 3D tissues/organs geometries comparable to what is found in vivo [57,59]. Current 3D bioprinting techniques can be divided into two categories: Nozzle-based (inkjet bioprinting and extrusion-based bioprinting) and Optical-based (laser direct-write bioprinting and stereolithographic bioprinting) [4]. Nozzle-based 3D bioprinting uses a nozzle to deposit bioink (usually a cell-laden material) onto a substrate. One benefit of this technique is the decreased costs and manufacturing time, but these previously mentioned benefits come at the expense of resolution [5,60,61,62]. In fact, resolution from 3D bioprinting can often be significantly less than photolithography and conventional 3D printing. Compared to the nozzle-based printing method, the optical-based bioprinting method transfers bioink onto a substrate by ejection or cross-linking with light-based material interactions [63]. The benefits of this method is reduced potential to damage the biomaterials used for ejection, especially cells, and the ability to create highly robust 3D patterns [64,65,66,67]. However, the disadvantages of 3D bioprinting are the cost is high, and there is a limitation on the materials that can be used.

An example of a device created using bioprinting is one created by Bertassoni et al., who constructed the microvessel networks using 3D bioprinting and GelMA hydrogel laden with mouse calvarial pre-osteoblasts cells [68]. Initially, agarose fiber (a naturally derived polysaccharide) was bioprinted as a template for the vascular structure. The hydrogel was polymerized around the template to provide a functional and perfusable micro-network after the template was removed by extracting it from the hydrogel. These micro-networks were designed to replicate the in vivo microvasculature. To help simulate flow conditions, human umbilical vein endothelial cells cultured as an inner lining. These microchannels can improve the functionality of mass transport, cellular viability, and differentiation, which can be used for further studies of physiology, pathology, and drug development and delivery with cardiovascular disease.

### 3.4. Computer Numerical Control Micromilling

Computer Numerical Control (CNC) milling allows for the accurate and repeatable creation of devices via the use of automation in material removal. Small scale CNC milling is also known as micro-milling. CNC micro-milling has gained attention as another alternative to fabricating lab-on-a-chip devices [69,70,71]. Although CNC milling is a near opposite production method when compared to 3D printing, it also compliments 3D printing well. Similar to 3D printing, micromilling uses a computer-aided design (CAD) file as a template for the milling machine to create. The use of CAD files allows for the cheap and fast production of custom as well as rapid prototyping [69]. Resolutions achievable using this method vary depending on the quality of the milling device and material used, but in general structures as thin as 3 μm can be created [72].

**Figure 5:**
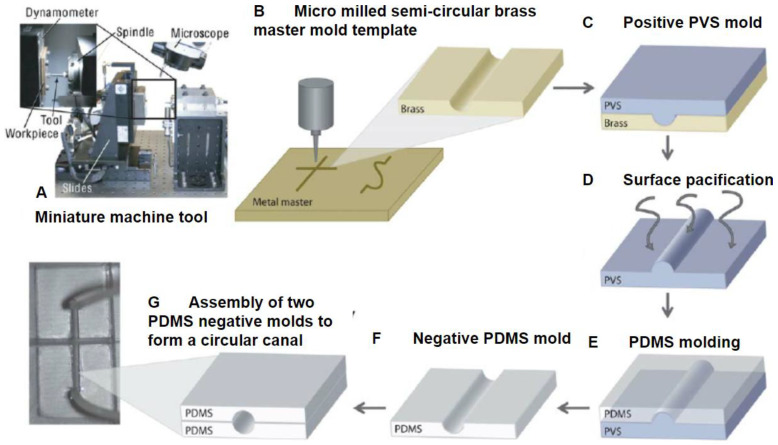
(**A**) Miniature machining tool used for micromilling (**B**) Micromilled master template for soft lithography (**C**) Using Poly-vinylsiloxane (PVS) to create a positive mold (**D**) Passivation of surface to prevent sticking (**E**) soft lithography with PDMS (**F**,**G**) assembly of two PDMS molds to create a channel. Adapted from Wilson et al. [71].

A clear advantage of micromilling is its ability to allow manipulation of materials with stiffnesses that are orders of magnitudes higher than other methods such as metal and glass, for example. In fact, a variety of geometries are achievable using these materials with specialized end mills, burrs, and bits utilized during the milling process. It is also important to note that if microscopy is to be performed utilizing a CNC-milled device, specialized mill heads should be used as this will affect the transparency of the device [72]. However, a significant limitation is the milling tool orientation as most CNC mills have the tool head arranged vertically above the material that is to be milled. This fixed, vertical tool orientation only allows for the top surface of the material to be milled, making it challenging to fabricate overhanging structures with a single piece of material if desired [70]. To alleviate this issue specialized tool heads and a five-axis milling machine can be utilized, but at a significantly increased cost. Another alternative solution that can be utilized is to mill the desired device out of two pieces of material and subsequently bonding them together. This technique can also by using soft lithography to bind milled geometries (Figure 5). For cardiovascular studies, micromilling provides a method of producing small structures such as microchannels and larger structures such as wells and housing components. Each method has its pros and cons when used alone but can offer significant advantages when combined [71].

## 4. Microfabrication Materials

### 4.1. Polydimethylsiloxane (PDMS)

Polydimethylsiloxane (PDMS) is one of the most commonly used materials in lab-on-a-chip fabrication, specifically during soft lithography due to its ease of use and low cost [73]. PDMS has been widely used in medical applications as it is biocompatible, non-toxic, and convenient to use [40]. PDMS is commonly used in photolithography and traditional 3D printing to provide a biocompatible cellular environment using a very straightforward process [9,74,75]. In general, the PDMS mixture (base with a cross-linking agent) is poured into a designed microstructure that is cured and molded. Since PDMS starts out as a liquid it can essentially create any structure conceivable through a mix of molds, printing, or milling. Li et al. mimicked the dimensions and network structure of microvessels using photolithography and PDMS, for example [76]. Additionally, devices constructed with PDMS provide long-term and continuous perfusion capability, which can be used to study various cardiovascular pathologies and physiologies. Marsano et al. presented a PDMS-based microfluidic device that could replicate the native myocardium structure and function found in vivo [77]. This device contained an array of hanging, cell-laden fibrin gels, and a pneumatic actuation system that could induce homogeneous uniaxial cyclic strains during cell culture. This closely mimicked the environment in the myocardium and could be further used to predict the signs of hypertrophic changes [77]. In general, the use of PDMS has provided an easy way to fabricate lab-on-a-chip devices, but the disadvantages of using this material are (1) gas bubbles can easily form during the curing process and (2) the PDMS device can be easily damaged due to the improper handling or hard instruments causing tearing or punctures.

### 4.2. Hydrogels

Hydrogels are often utilized since they can be used to model particle diffusion across membranes and therefore study permeability [78,79,80]. This is an important property for studying cardiovascular pathologies, especially those related to the vasculature since functioning as a selectively permeable barrier is among the most important functions of the vasculature [78,81,82]. Furthermore, hydrogel materials offer advantages similar to PDMS, such as biocompatibility, flexibility, and relatively low cost [83,84]. In general, hydrogel stiffness can be fine-tuned to stiffness much softer and physiologically relevant than PDMS. It also shares the same advantage in that it can also be used with soft lithography and can be integrated with PDMS devices as well. Annabi et al. successfully constructed a cell-compatible hydrogel layer inside a PDMS microfluidic device that could be used for studies using elastic tissues (within the use of cardiomyocytes) which could be further used for cardiovascular drug screening [85]. However, it should be noted that when combining these two materials it can be difficult to control the thickness of the hydrogel layer if simply using it as a coating. Another note is that gas bubbles are prone to form on the surface of the hydrogel during the curing process this can be alleviated by degassing the gel when in liquid form.

### 4.3. Gelatin Methacryloyl (GelMA)

Gelatin Methacryloyl (GelMA) is commonly used in 3D printing due to its excellent biocompatibility, cytocompatibility, and the ability to precisely control of substrate stiffness making this material suitable for a host of biological experiments that may involve cell proliferation, cell migration, and cell–cell and cell–substrate interactions [86,87,88,89,90,91]. Kitsara et al. developed a microfluidic device to mimic a native 3D structure of endothelialized myocardium using a 3D bioprinting technique that utilized GelMA [92]. A microfibrous scaffold was bioprinted using the encapsulation of human umbilical vein endothelial Cells (HUVECS) in alginate-GelMA hydrogel to induce the migration of the endothelial cells and form a confluent, endothelial monolayer. This bioprinted endothelized scaffold was then seeded with cardiomyocytes to induce the formation of a myocardium, which could structurally resemble the native structure of myocardium found in vivo for further studies of various pathology and physiology related to cardiovascular. In general, the use of GelMA material in 3D bioprinting has provided an ability to closely mimic the 3D tissue structure found in vivo within the use of various cell types encapsulated directly. However, GelMA is more fragile compared to the previously mentioned materials and therefore are more prone to fracture and breakage.

## 5. Cardiovascular Pathology-on-a-Chip Applications

### 5.1. Drug Screening

Cardiovascular pathology-on-a-chip devices have the benefit of being able to conduct in vitro drug-delivery testing using cellular and tissue models as it allows for spatial and temporal control of drug delivery. Cardiovascular pathology-on-a-chip has several advantages over traditional animal model testing and this goes beyond just cost. First, cardiovascular pathology-on-a-chip can use human cells and tissues. The use of human cells allows for the testing of medication without the error and potential drug compatibility issues associated with using animal models [2,93,94]. Nineteen percent of compound withdrawals are due to concerns about negative effects on cardiovascular health [95]. Second, the cardiovascular pathology-on-a-chip platform can utilize cells from different demographics, which is important as it has been well-established that the efficacy of certain medications varies among different patient demographics [10,96]. Finally, this testing platform allows for high-throughput testing of standard properties such as toxicology, gene expression, and cell apoptosis, for example [31,95,97]. One such cardiovascular pathology-on-a-chip drug-screening study examined the effects of various drugs on Cardiac fibrosis and is described below.

**Figure 6:**
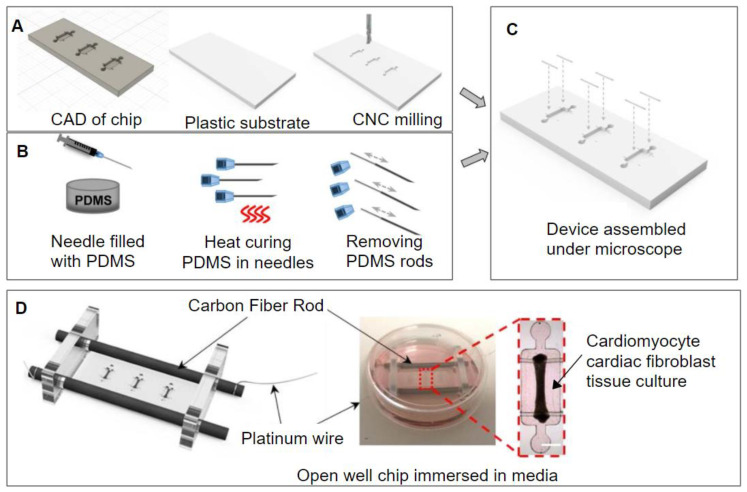
(**A**) using a computer-aided design (CAD) to micromill the microwells out of plastic (**B**) using 27-gauge needles as a mold for the creation of uniform PDMS rods. (**C**) the assembly of the two pieces to make the open well device. (**D**) adding carbon fiber rods and platinum wire to apply a voltage across the open well tissue culture. Adapted from Mastikhina et al. [31].

Mastikhina et al. created a lab-on-a-chip device that could measure the effects of various therapeutic molecules on cardiac fibrosis. This chip was CNC milled out of acrylic or polystyrene and PDMS rods were molded using 27-gauge needles (Figure 6) [31]. These rods would support the tissue co-culture that would be suspended by them. The tissue co-culture was grown using human induced pluripotent stem cell-derived cardiomyocytes (hiPSC-CMs) and cardiac fibroblasts. Fibrotic tissue was created for comparison to healthy control. This was carried out by treating the cardiac fibroblasts with TGF-β1. The fibrosis-on-a-chip device was used to determine the effects of pirfenidone, a drug used to treat cardiac fibrosis, and Losartan and Carvedilol, two antihypertensive drugs on the multicellular system. The relative change in fibroblast collagen deposition was by the fibroblasts monitored using second harmonic imaging and α-SMA was observed as well. In addition, the amount of brain natriuretic peptide (BNP) secreted and gene expression was measured as well [31]. The device showed the drugs mentioned above to have the following effects, pirfenidone reduced tissue stiffness and BNP secretion and improved passive tissue tension. losartan and carvedilol significantly decreased BNP secretion. Other parameters did not see a significant change. 

### 5.2. Electro-Mechanics

Cardiomyocytes are unique muscle cells due to their ability to spontaneously depolarize. Spontaneous depolarization occurs when cardiomyocytes produce action potentials similar to other muscle cells, but they do not need to receive a signal from the central nervous system to autonomously depolarize [14,98]. This depolarization causes other cardiomyocytes to depolarize, leading to a depolarization signaling cascade. This cascade of depolarizing cells creates a front that can propagate throughout the heart muscle, allowing cardiomyocytes to beat synchronously even when not stimulated by an external source [98,99,100]. In addition, the ability of cardiomyocytes to autonomously depolarize allows for the study of the pacing and propagation of this electrical signal across a culture of cardiomyocytes and the study of heart fibrillation. Another aspect that can be studied is the force that cardiomyocytes exert when they contract. To do this electrical stimulation can be used to cause all the cardiomyocytes to contract at a predetermined rhythm. Both the propagation of depolarization and the strength of cardiac contraction can and often are altered by disease states and medications [31,101,102].

**Figure 7:**
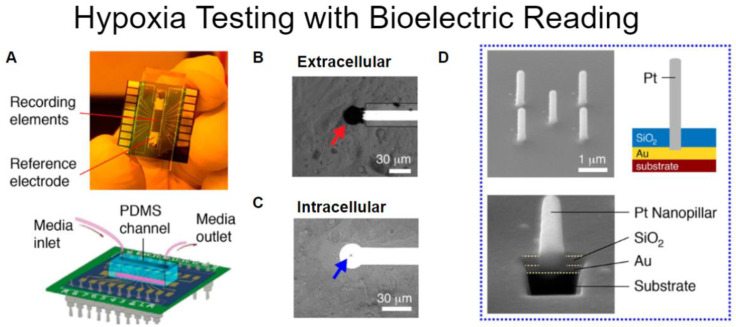
(**A**) Lap on a Chip made to measure the bioelectrical signals that cardiomyocytes produce. Visual reference top and schematic bottom. (**B**) Image of an extracellular element that cells grow on top of, in black. (**C**) a small arrangement of five intracellular platinum elements for cells to grow around. (**D**) Top left an arrangement of five platinum nanopillars for measurement. Bottom left cut out showing platinum nanopillar penetration into the Au layer. Right cross-section of single platinum nanopillar. Adapted from Liu et al. [14].

First, looking at measuring the electrical signals of cardiomyocytes, the propagation of depolarization can be monitored via microelectrode arrays. A device described by Liu et al. uses an electrode array that has cardiomyocytes grown on its surface to observe how oxygen concentration affects cell function. The electrode array can gather data from electrical signals by monitoring the voltage changes between any two electrodes. Liu et al. Created a lab-on-a-chip with two different types of electrodes that could take intracellular and extracellular readings (Figure 7). A microfluidic canal on the device allowed for the control of oxygen content in the media and to the cells. The intracellular electrodes were used to measure the action potential of cardiomyocytes in normal and hypoxic conditions. It was discovered that lowering the oxygen content in the media to the cardiomyocytes created fibrillation in the cells. The extracellular electrodes were able to map the propagation of depolarization and provide a visualization of fibrillation along with recording the number of depolarization cycles and how the rate changed under hypoxic conditions over time. 

**Figure 8:**
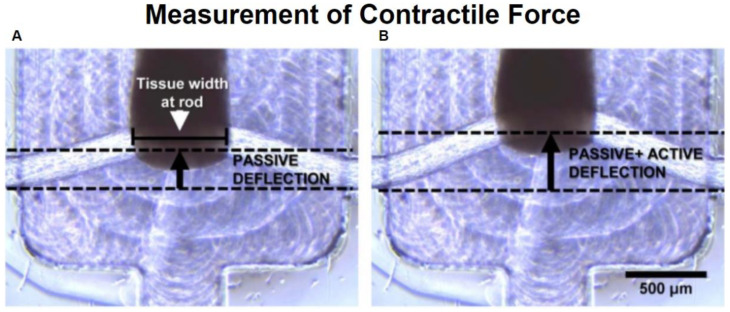
Measurement of pulsatile strength using a PDMS rod. By measuring the deflection at rest (**A**) against the deflection during stimulated contraction (**B**) the force exerted by the tissue can be measured. Adapted from Mastikhina et al. [31].

**Figure 9:**
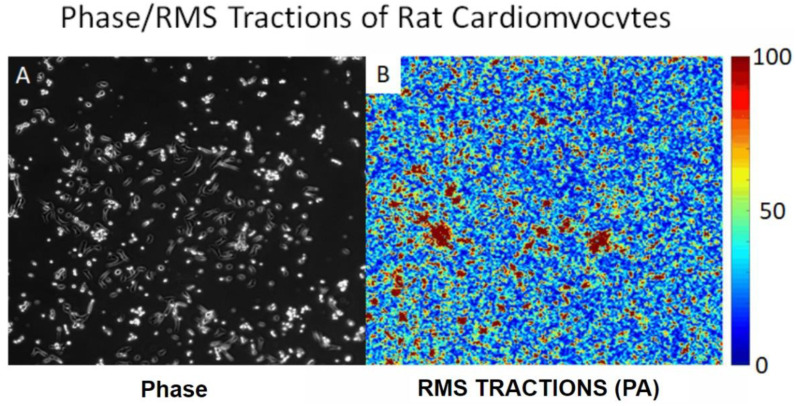
(**A**) Phase image of rat cardiomyocytes on 1200 kPa stiffness hydrogel. (**B**) 2D map of tractions exerted by the cells on their substrate.

The fibrosis-on-a-chip described above by Mastikhina et al. was also able to be used to determine the contractile force of normal and fibrotic cardiac tissue. This was carried out by applying electrical stimulation to the heart tissue at voltages comparable to what these tissues would experience in vivo. This allowed for monitoring of two indicators of the heart tissues ability to function; (1) The determination of how fibrosis and the drugs Losartan and Carvedilol affects the sensitivity of the cardiomyocytes to depolarize and (2) the predictable pace of the pacemaker allowed for the contraction of the whole tissue so that the contractile force could be measured. The measurement of contractile force was measured by monitoring the deflection of two PDMS rods (Figure 8). The heart culture was grown so that a strip of tissue spanned and connected the two rods. As the heart contracted, it would deform the rods. The amount of deflection of these rods would then be measured and using a custom calibration formula the contractile force exerted by the tissue could be calculated [31]. Another similar technique using deformation to calculate the force cardiomyocytes exert is traction force microscopy. Traction force microscopy consists of attaching cells to a deformable substrate. Cells can then be stimulated into contraction using a similar method described above. As the cells contract, they exert forces on their underlying substrate known as tractions [103,104,105]; an example of tractions exerted by contracting cardiomyocytes is shown in Figure 9.

### 5.3. External Mechanical Forces

Major pathological factors that contribute to the initiation and progression of cardiovascular diseases include the formation and maturation of cholesterol-rich plaque build-up, thrombosis, and/or stenosis in the blood vessels [15,18,106]. In addition, these pathologies are often related to the hemodynamic forces induced by blood flow on the vessel wall [24,107]. In general, the influence of the fluid flow on cardiovascular diseases has been studied both in vivo and in vitro. Below we present studies examining the influence of external forces on cardiovascular diseases.

**Figure 10:**
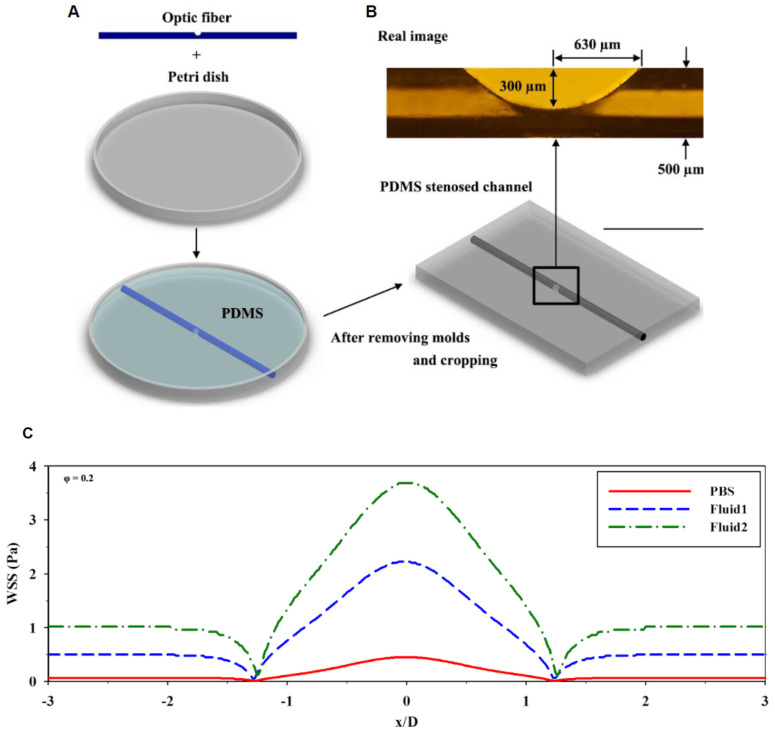
(**A**) Creation of the PDMS stenosed microchannel using a polymethylmethacrylate optic fiber and a petri dish. (**B**) Illustration of the fabricated PDMS stenosed channel (bottom) and actual image (top) captured using a microscope lens. (**C**) the distribution of the Wall Shear Stress (WSS) for the stenosed wall with Phosphate-Buffered Saline (PBS), fluid 1 (consisted of 79.1% (*v*/*v*) distilled water, 20.9% (*v*/*v*) glycerol, and 0.21 g/L of xanthan gum for a blood analog fluid [31]; fluid 2 comprised 0.42 g/L of xanthan gum under the same volumetric ratio of water and glycerol), fluid 2 (comprised 0.42 g/L of xanthan gum under the same volumetric ratio of water and glycerol). Adapted from Hong et al. [110].

**Figure 11:**
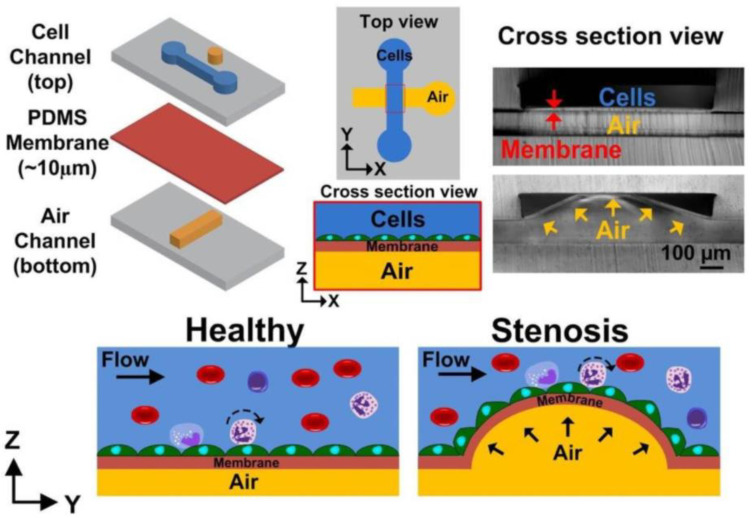
Schematic of the PDMS-based microfluidic device consisted of a cell channel (**top**), thin PDMS membrane (**medium**), and air channel (**bottom**). Air pumped into the bottom channel deflects the PDMS membrane upwards to create a 3D constriction (artificial atherosclerosis plaque) in the stenosis region of the top channel. Adapted from Menon et al. [8].

Stenosis of a blood vessel can occur due to abnormal narrowing of the blood vessel caused by the deposition of cholesterol and other lipids on the interior of the vessel wall [107,108], affecting the fluid flow behavior within the vessel and potentially becomes directly involved in the progression of atherosclerosis [106,109]. It is important to understand the relationship between the flow behavior and stenosis formation. Hong et al. [110] designed a simulation of stenosis with a microfluidic chip using PDMS and polymethylmethacrylate optic fibers (Figure 10A). The main goal of this device was to measure and analyze the fluid velocity profiles and fluid shear stress on the walls inside a stenosed channel. This was performed using a non-Newtonian water and glycerol fluid solution and Newtonian phosphate-buffered saline (PBS) fluid solution [110]. This device allowed the study of different fluid flow behaviors and their relationship with fluid shear stress at the stenosis regions. This study revealed a Newtonian fluid with higher viscosities to increase fluid shear stress around the stenosis region (Figure 10B), which could subsequently induce the risk for lesion rupture and damage the surrounding tissue [110,111,112]. Menon et al. constructed a 3D stenosis tunable microfluidic chip (which could mimic the atherosclerosis plaques condition in the blood vessel) using PDMS and 3D air bump technique (Figure 11) [8]. This air bump was created by placing a flexible PDMS membrane between two canals. One canal to carry fluid flow for cell culture and a pneumatic canal to apply pressure and cause the PDMS to bulge, creating an adjustable stenosis region. This lab-on-a-chip allowed for the analysis of significant changes between the various flow profiles at a stenosis region and observe how stenosis impacts vascular inflammation and leukocyte–endothelial cell interactions during the development of atherosclerosis. It was found under low shear stress for stenosis conditions (especially the 80% constriction stenosis condition), there was a significant increase in monocyte adhesion (a primary factor involved in the progression of atherosclerosis) [113]; which can change endothelial barrier function by further increasing chances for thrombus formation due to accelerated plaque formation [26]. 

**Figure 12:**
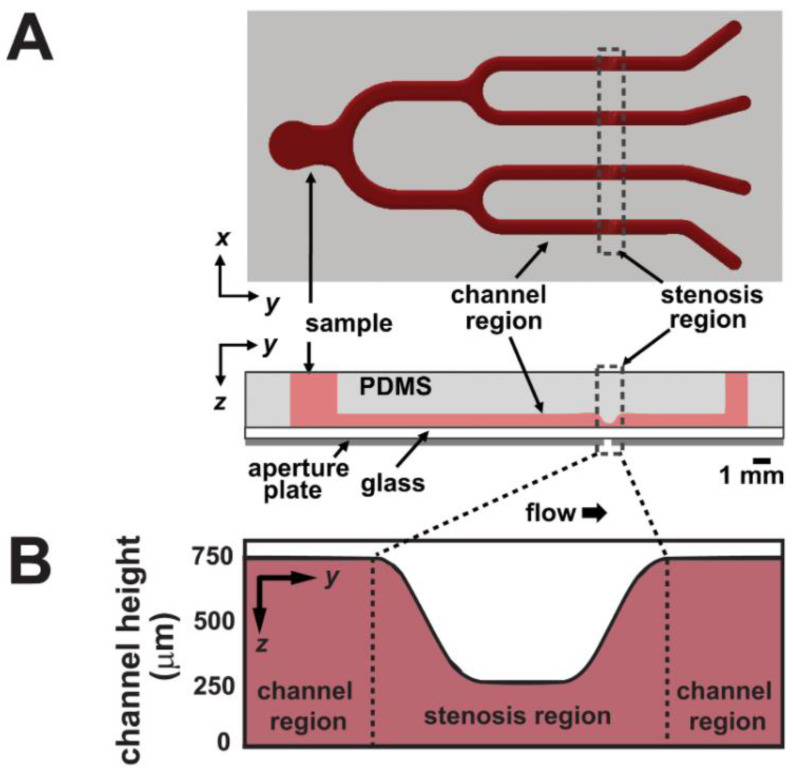
(**A**) schematic images of the microfluidic chip with four identical stenosis regions. (**B**) cross-section of the stenosis region. Adapted from Li et al. [22].

**Figure 13:**
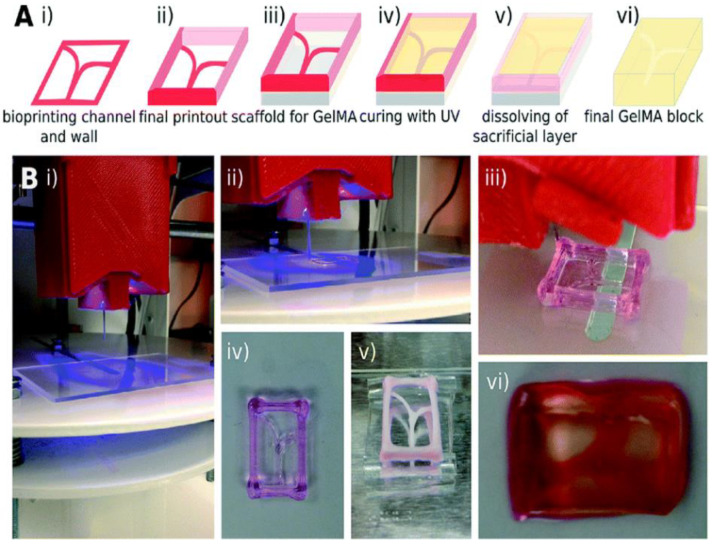
(**A**) Schematic of the bioprinting process: (**i**,**ii**) bioprinting the Pluronic template; (**iii**) dried out the bioprinted template on a PDMS support; (**iv**) filled with GelMA inside the channel and cured with UV cross-linking; (**v**) dissolution of the sacrificial channels and frame; (**vi**) the final construct with hollow channels coated with GelMA. (**B**) Photographs showing the experimental steps corresponding to the sacrificial bioprinting process illustrated in (**A**). Adapted from Zhang et al. [7].

Fluid shear has been suggested by many groups to be a contributing factor to the initiation and progression of thrombosis. In fact, fluid shear can lead to platelets along with other cellular materials to aggregate excessively along the interior of the vessel wall; forming a thrombus that can partially or completely restrict blood flow through the vessel [114]. Therefore, understanding the influence of different fluid shear stress rates involved in thrombus formation is important in studying the pathology of thrombosis. Li et al. constructed a microfluidic chip that contained four identical stenoses (Figure 12). To create this, fibrillar equine collagen was used to fill the inside stenoses channels to initiate the platelet adhesion to stimulate a disease condition that could cause thrombosis. This device allowed for the measurement of the time it took for a thrombus to form, to cause occlusion, and for the thrombus to detach as a function of fluid shear stress rates and antiplatelet therapeutic concentrations [22]. Results revealed higher shear rates to have significant effects in both thrombus occlusion, thrombus detachment, and antiplatelet therapies. Under Cetyl-salicylic acid (ASA) therapy, thrombi formed at high shear rates were four times more prone to detached compared to other conditions, and Eptifibatide therapy could reduce occlusion when controlling the shear rates with effective dose concentration. In a similar study, Zhang et al. used a 3D bioprinting technique to mimic the condition that causes thrombosis. This was performed with a lab-on-a-chip by embedding human umbilical vein endothelial Cells (HUVECS) in alginate-GelMA hydrogel. This hydrogel was molded around a bioprinted Pluronic template (Figure 13) [7]. This template was made via a bioprinted canal and a containing mold made from sacrificial Pluronic material. GelMA was then cured and the sacrificial Pluronic was then dissolved to leave a usable canal for flow studies. This microfluidic chip allows the study of thrombosis and fibrosis by infusing whole human blood through the microchannel following thrombus activation (10 vol.% 0.1-M CaCl2 in DPBS treatment). Results indicate the disturbed flow profiles within the selected perfusion flow rates successfully formed thrombus at the bifurcation region inside the microfluidic channel. Additionally, the gel encapsulated fibroblasts and endothelial cells were observed to migrate into the thrombosis resembling the in vivo scenarios of fibrosis/thrombosis. The results demonstrated this lab-on-a-chip to be a reasonable analog for further personalizing vascular disease studies, including drug screening for vascular thrombosis.

## 6. Research Gaps and Future Outlook

There are several research gaps associated with lab-on-a-chip cardiovascular-based technologies. One such gap is the lack of physiological and pathological-based testing of various demographic groups. Demographic-based testing within lab-on-a-chip technologies is important, as the effectiveness of various therapies was demonstrated to vary based on demographic group. The point mentioned above represents a gap in the field, but in revealing this gap, we also suggest the tremendous future potential for the field. Lab-on-a-chip has the advantage of being able to utilize cells from a wide variety of demographics that allows for the study of underrepresented populations, which is more cost-effective than the alternative in vivo studies. Since the effectiveness of medication can vary based on demographics, the ability to test cells from specific demographics is also an important tool for pharmacology [10,94,96,101,115,116]. In addition, lab-on-a-chip allows for the simulation of various cardiovascular disease conditions at the cellular level that can mimic behavior at the tissue and potentially the whole organ level [7,8,10,31,94,96,101,110,114,115,116]. Drug screening for cardiovascular disease is another potential application where lab-on-a-chip will no doubt be useful for the future as the lab-on-a-chip testing platform can allow for cellular-based drug toxicity studies [101,115]. In addition, lab-on-a-chip technologies have advanced and evolved to organ-on-a-chip studies where organ-level functions can be mimicked at the benchtop. Though true, the ability to reproduce a completely functional “organ” with all the respective cell types remains a challenge in the field. A last future outlook in the field consists of the ability to use lab-on-a-chip technologies to study the complex interplay that exists between the cardiovascular system as well as other complementary systems such as the respiratory system and neurological system [32,33,34,63,64,93,117].

## 7. Conclusions

The utilization of cardiovascular pathology-on-a-chip devices has provided the opportunity of a more convenient way to study the pathology of cardiovascular diseases at a reduced cost and level of complexity when compared to in vivo studies. This platform allows for drug screening with variable amounts of medication administered via microfluidic channels, making it a valuable resource for research and clinical studies [1,3]. Looking at the common fabrication techniques (photolithography, CNC milling, 3D printing) and materials used in the construction of cardiovascular pathology-on-a-chip, the advantages of this platform include being able to reproduce realistic geometry similar to the human body and allowing for the use of human cells and tissues. An additional advantage is the ability to grow cells related to specific disease states such as coronary artery disease and cardiac fibrosis. Furthermore, the materials described above are relatively cheap and readily available and accessible.

Similar to lab-on-a-chip being used to study the pathology of cardiovascular diseases, a new variant on the idea has become an interest. Body-on-a-chip is the next step in the growth of the lab-on-a-chip-platform. Body-on-a-chip integrates multiple “organ-on-a-chips” in one biological system that can replicate the entire human body. The idea of this new research topic has provided some guidance in the future to study, for example, to extend and validate the human pharmacokinetic and pharmacodynamic models during the drug discovery and development processes [32,33]. Additionally, body-on-a-chip has provided an opportunity to study the disease pathology and physiology in the early stage, which could greatly help for treatment processing [34]. However, accompanying multiple organ-on-a-chip in one biological system is difficult and challenging since it will ideally replicate an entire human system from in vivo to in vitro. The overall stability and viability of the multi-organ network could become an issue; issues with culture medium requirements to sustain many cell types, and the volume of material cells and media required are the example limitations there still exist. Therefore, future research and studies are needed to replicate the entire human biological system using multiple organ-on-a-chip, but its potential to advance the field is promising.

In summary, cardiovascular pathology on-a-chip allows for a better understanding of the physiology and pathology of the human vasculature and their related cardiovascular diseases in a convenient, cheap, and accurate package.
